# Cranberry Extract Ameliorates Diabetic Cognitive Impairment in Rats *Via* LncRNA GAS-5 Downregulation and Pyroptosis Pathway Inhibition

**DOI:** 10.1007/s11481-025-10199-1

**Published:** 2025-04-21

**Authors:** Mariam Ali Abo-Saif, Amany E. Ragab, Iman M. Talaat, Maha Saber-Ayad, Amera O. Ibrahim, Hend Mostafa Selim

**Affiliations:** 1 Biochemistry Department, Faculty of Pharmacy, Tanta University, Tanta, 31527 Egypt; 2 Department of Pharmacognosy, Faculty of Pharmacy, Tanta University, Tanta, 31527 Egypt; 3 Clinical Sciences Department, College of Medicine, University of Sharjah, Sharjah, 27272 United Arab Emirates; 4 Research Institute for Medical and Health Sciences, University of Sharjah, Sharjah, 27272 United Arab Emirates; 5 Pathology Department, Faculty of Medicine, Alexandria University, Alexandria, 21131 Egypt; 6 Department of Pharmacology, College of Medicine, Cairo University, Giza, 11956 Egypt

**Keywords:** LncRNA GAS-5, Growth-arrest-specific (GAS)-5, Pyroptosis, Caspase 1, Gasdermin D, Cranberry extract, Diabetes-induced cognitive impairment, GDNF

## Abstract

The pathophysiology of diabetes-induced brain injury involves pyroptosis, an inflammatory programmed cell death. This study aimed to investigate the potential protective effect of cranberry extract (CE) against diabetes-induced brain injury. Type 1 diabetes was induced by intraperitoneal injection of streptozotocin in rats. Brain tissue samples were investigated for biochemical determination of the reduced glutathione (GSH), superoxide dismutase (SOD), and malondialdehyde (MDA), and the quantitative RT-PCR for the gene expression of glial cell-derived neurotrophic factor (GDNF), lncRNA GAS-5, and pyroptosis markers. ELISA was used to determine the caspase-1 level and immunohistochemical staining for assessing IL-1β. Prophylactic dosing of the CE in diabetic rats improved cognitive behavior and significantly suppressed MDA concentration, pyroptosis genes expression (gasdermin D and caspase 1), and lncRNA GAS-5. In addition, CE significantly elevated GSH concentration, SOD activity, and gene expression of GDNF and markedly reduced IL-1β positive stained cells score in the brain. Phytochemical characterization of the CE by FT-IR and UPLC-PDA-MS/MS revealed cyanidin arabinoside, procyanidins, quercetin, and isorhamnetin as key components. CE protects against diabetes-induced cognitive dysfunction in rats by targeting redox-related signaling pathways and inducing an anti-inflammatory effect. LncRNA GAS-5 downregulation and pyroptosis pathway inhibition may contribute to its beneficial effects, suggesting its therapeutic potential.

## Graphical Abstract

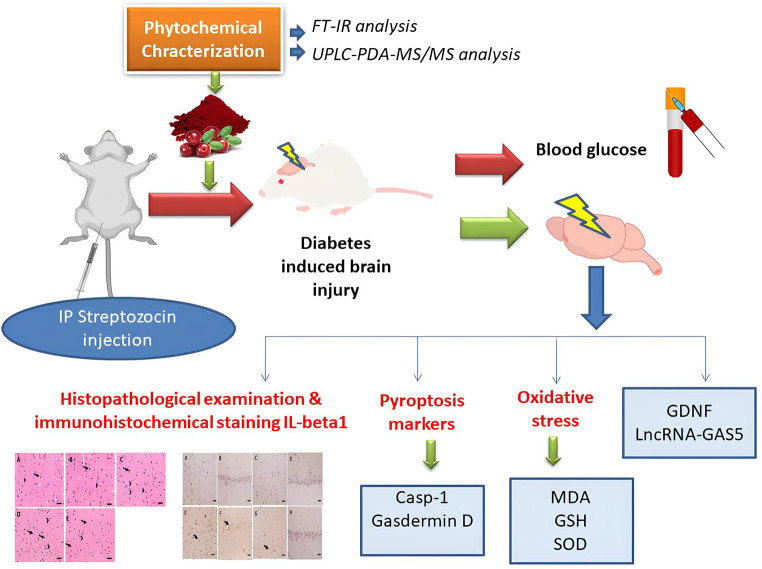

## Introduction

Type 1 diabetes is a metabolic auto-immune disease characterized by hyperglycemia due to the inability to produce insulin. The high blood glucose level in diabetic patients contributes to producing reactive oxygen species (ROS) and microvascular abnormalities that affect most body organs. One of the most affected organs is the brain (Chen et al. 2018; Rom et al. 2019). Thus, as a consequence of type 1 diabetes, the brain reflects signs of cognitive impairment/dementia and injury (Chen et al. 2018; Rom et al. 2019).

Pyroptosis is an inflammatory programmed cell death provoked as a response to cellular damage, oxidative stress, inflammation, or metabolic imbalances (Xue et al. 2019). Pyroptosis is mediated by various inflammasomes that can identify exogenous or endogenous danger signals. The trigger of pyroptosis is strongly linked to the formation of the nucleotide-binding domain (NOD)-like receptor protein 3 (NLRP3) inflammasome complex. Oligomerization of NLRP3 inflammasome boosted the activation of caspase 1, which subsequently converts the inactive proinflammatory cytokine IL-1β into its active form. Caspase 1 uses gasdermin D to facilitate the release of the activated IL-1β (Zheng and Li 2020).

Oxidative stress and hyperglycemia associated with type 1 diabetes have been implicated in activating various biological pathways, including pyroptosis [1, 2, 5,] [6,] (Che et al. 2020). Moreover, metabolic imbalances in diabetes due to hyperglycemia can activate the NLRP3 oligomerization and the subsequent related events that cause cell injury and death (Ruan et al. 2022). In addition, previous studies showed that activation of pyroptosis in diabetes interrupts the blood-brain barrier, causing cognitive impairment and dementia (Liu et al. 2022; Scassellati et al. 2021). Therefore, the inhibition of pyroptosis has a neuroprotective effect on the diabetic brain (Che et al. 2020).

Long non-coding RNAs (lncRNAs) are a group of RNA transcripts that cannot encode protein. LncRNAs are embroiled in multiple pathological mechanisms and are implicated in different diseases (Khorkova et al. 2015). Growth arrest-specific 5 (GAS-5) is a lncRNA essential for regulating various diseases, including diabetes and cancer (Shi et al. 2019). GAS-5 is correlated with NLRP3 activation and the progression of cognitive impairment in Parkinson’s disease (Xu et al. 2020).

Until now, there has been no specific treatment for diabetes-induced brain injury and cognitive impairment. The available protective strategies involve using antioxidant and anti-inflammatory agents (Xiao et al. 2022).

Colored fruits contain many compounds, such as organic acids, terpenes, and polyphenols, which exhibit antioxidant and anti-inflammatory activities and are of remarkable biological value (Suriyaprom et al. 2022). For example, cranberry (*Vaccinium oxycoccos* L., family Ericaceae) contains vitamins E and C. It is rich in different polyphenols, including anthocyanins and proanthocyanidins, which show remarkable antioxidative activity (Shimizu et al. 2019). Anthocyanins, the main compounds accumulated in CE, have potent anti-neuroinflammatory activity and could prevent and treat various brain disorders (Henriques et al. 2020). On the other hand, Pourshahidi et al. (Pourshahidi et al. 2020) revealed that anthocyanins inhibit the pyroptosis pathway in squamous cell carcinoma, which is attributed to its anti-cancer effect.

Cranberry contains polyphenols that have beneficial properties in modulation of both glucose and lipid metabolism (Li et al. 2024). Previous studies supported its effect demonstrating that cranberry extract significantly improved lipid profile, decreased fasting blood glucose and homeostatic model assessment of insulin resistance (HOMA-IR) levels (Niesen et al. 2022; Peixoto et al. 2018; Yung et al. 2013). Moreover, it was previously reported that the polyphenol-rich cranberry extract possess strong antioxidant activity that controls oxidative stress in several organs with the most noticeable effect in the brain(Peixoto et al. 2018; Yung et al. 2013; Flanagan et al. 2022). Furthermore, it was reported previously that sustained intake of cranberry produced significant improvements in memory and neural function in older adults (Flanagan et al. 2022).

This study aimed to investigate the potential protective effect of CE against diabetes-induced brain injury and cognitive impairment in rats and to determine the possible underlying molecular mechanism alongside the phytochemical characterization of the extract constituents. This work provides insight into potential preventive and therapeutic targets for managing diabetes-induced brain injury. We demonstrate that the protective effect is mediated through the downregulation of lncRNA GAS-5, thus inhibiting pyroptosis.

## Materials and Methods

### List of Chemicals and Kits

- The CE was obtained from Shaanxi Jiahe Phytochem Co. (Xi’an, China).
- Streptozotocin (Sigma Aldrich, St. Louis, USA).

− 0.1 M sodium citrate buffer (Sigma Aldrich, USA).

- Cold saline (pH = 7.4).
- Phosphate-buffered saline (PBS, pH 7.5) (Sigma Aldrich, USA).

− 10% neutral buffered formalin (Bio-optica, Italy).

- Paraffin, xylene, ethanol.
- Hematoxylin and eosin solution (Sigma Aldrich, USA).
- MDA, GSH, and SOD activity assay kits (Biodiagnostic, Cairo, Egypt).
- miRNeasy® mini kit (Qiagen Co, Hilden, Germany).
- Two-step RT-PCR kit (Qiagen Co, Hilden, Germany).
- QuantiTect SYBR Green I PCR (Qiagen Co, Hilden, Germany).
- The primers were purchased from Willowfort Co. (Birmingham, England).
- Rat caspase 1 ELISA kit (MyBioSource, California, USA).
- Anti-IL-1β (1:200) (Sigma Aldrich, St. Louis, USA).

### Plant Material

The CE was obtained from Shaanxi Jiahe Phytochem Co. (Xi’an, China) as a dry powder of water and ethanol extract of cranberry.

### Phytochemical Characterization of the Extract

#### FT-IR Analysis

The CE was analyzed as a KBr disc using an FT/IR-6100 spectrophotometer (Jasco Co., Tokyo, Japan).

#### UPLC-PDA-MS/MS Analysis

The CE was characterized using UPLC-PDA-MS/MS as described before (Pei et al. 2024). A liquid chromatography Nexera-i LC-2040 system (Shimadzu, Kyoto, Japan) connected to a C18 column (UPLC Shim-pack Velox, 2.1 × 50 mm; 2.7 μm particle) was used. The preparation of the sample and the mobile phase gradient were done as previously reported (Assar et al. 2021a, b; Ragab et al. 2022). The compounds were detected through a photo diode array (PDA) detector of the type of LC-2030/2040 and a triple quadrupole mass spectrometer (LC-MS 8045) equipped with an electrospray ionization (ESI) source in a negative mode (Shimadzu, Kyoto, Japan) (Assar et al. 2021a, b; Ragab et al. 2022).

### Biological Evaluation

#### Animals

Thirty six male Wistar rats (aged two months and weighing about 220–250 g) were purchased from the National Research Center (Cairo, Egypt). Rats were supplied with a standard pellet chow and allowed free access to water and diet. Animals were housed for two weeks for acclimatization. The study was conducted according to the Guidelines for the Care and Use of Laboratory Animals approved by the Research Ethical Committee, Faculty of Pharmacy, Tanta University, Egypt (ethical approval code: TP/RE/11/22P-0060).

#### Experimental Protocol

After acclimatization, rats were randomly divided into four groups (*n* = 8) as follows: Group I; negative control (NC, healthy rats), Group II; CE only treated (healthy rats treated with the CE), Group III; positive control (PC, diabetic rats), and Group IV; diabetes treated (diabetic rats pre-treated with the CE).

For type 1 diabetes induction, a single i.p. dose of 60 mg/kg streptozotocin (Sigma Aldrich, St. Louis, USA) was dissolved in 0.1 M sodium citrate buffer and injected into each rat. The development of type 1 diabetes took 72 h after streptozotocin dosing. For the negative control group, rats were injected with a single i.p. dose of the vehicle (0.1 M sodium citrate buffer 0.25 mL/kg) (Babu and Srinivasan 1997).

Blood glucose level was measured using a blood glucose meter Accu-Chek Performa (Roche Diagnostics, Indianapolis, USA) *via* tail vein puncture. Only those rats that showed blood glucose levels above 16.7 mmol/L were considered diabetic and selected for further research(Babu and Srinivasan 1997).

Groups III and IV were given 1 mL of the CE orally at a dose of 250 mg/kg/day for eight consecutive weeks. While rats in groups I and II were given 1 mL of the vehicle (distilled water) orally for eight consecutive weeks (Hussain et al. 2017).

At the end of our experiment, the random blood glucose levels were determined, as well as the rats’ total body weight; then, rats were fasted for 16 h and were anesthetized by isoflurane (El-Nasr Pharmaceutical Co., Egypt). All rats were anesthetized with isoflurane (3.5% for induction and 2.5% for maintenance). Afterwards, blood was quickly withdrawn from the inferior vena cava of rats and then rats were sacrificed in the form of an immediate removal of the heart.

#### Brain Sample Collection

After the rats’ sacrifice, the brain was removed and washed immediately with cold saline (pH = 7.4). Then, each brain was cut into two portions. The hippocampal and cerebral cortices of rats in the first portion of different groups was fixed in 10% formaldehyde for histopathological assessment and immuno-staining of interleukin I beta (IL-1β). The other brain portion was kept at − 80 °C for further biochemical determination of the reduced GSH, SOD, and MDA, and the quantitative RT-PCR for the gene expression of GDNF, lncRNA GAS-5, and pyroptosis markers (gasdermin D and caspase 1).

#### Morris Water Maze (MWM) Test

The MWM test was carried out to evaluate the rats’ cognitive functions and memory retention (Yadav et al. 2017). Rats of each studied group were released in a large glass pool (50 cm × 75 cm in diameter, 60 cm in depth, and maintained at 25 ± 1 °C) with four corners. A platform was immersed about 2 cm below the surface of the water. Rats were allowed to find the platform in clear water within 1 min daily for five consecutive days. On day six, the rats were allowed to swim in the pool without the platform for 1 min for the probe test. Then, rats were tested to find the platform in cloudy water with milk powder. The tested rats were placed in the cloudy water facing the pool wall at one of the four starting points. Each rat was allowed to perform three trials every day. The cognitive behavior of the rat during the trial sessions was expressed by the time (sec) required for the rat to reach the targeted hidden platform (escape latency time). The mean of the three test trials for each rat per day was calculated for 4 days.

#### Determination of MDA and GSH Levels and SOD Activity

The brain tissue was used for the colorimetric measurement of MDA, GSH, and SOD activity using MDA, GSH, and SOD activity assay kits (Biodiagnostic, Cairo, Egypt), respectively. The brain tissues were homogenized in phosphate-buffered saline (PBS, pH 7.5) using a polytron homogenizer (PT 3100, Kinematica, Luzern, Switzerland). After that, the homogenate was centrifuged at 4000 rpm for 15 min at 4 °C, and the supernatant was collected to be used for the colorimetric measurements as described by the manufacturer.

#### RNA Extraction and qRT-PCR for the Assessment of the Gene Expression of GDNF, GAS-5, Gasdermin D, Caspase 1, and GAPDH

Total RNA was extracted from the brain tissue using the miRNeasy® mini kit (Qiagen Co, Hilden, Germany). The cDNA was prepared from the extracted RNA using a two-step RT-PCR kit (Qiagen Co, Hilden, Germany). The PCR was carried out using a QuantiTect SYBR Green I PCR (Qiagen Co, Hilden, Germany) by 10 s denaturing at 95 °C, 15 s annealing at 55 °C, and 25 s extension at 75 °C. The primers were purchased from Willowfort Co. (Birmingham, England). The sequence of the primers was: GDNF (NM_019139.2) forward (5′- GAC GTC ATG GAT TTT ATT CAA GCC ACC- 3′) and reverse (5′- CTG GCC TAC TTT GTC ACT TGT TAG C- 3′)(Ahmadiantehrani et al. 2014), GAS-5 (NR_002704.1) forward (5′- AGC CAG AAA ATG GGA TGG TGG- 3′) and reverse (5′- ACT GCA CTG TCC ACT TGT CA- 3′)(Chen and Zhang 2021), gasdermin D (NM_001400993.1) forward (5′-CCA ACA TCT CAG GGC CCC AT-3) and reverse (5′-TGG CAA GTT TCT GCC CTG GA-3′)(El-Shaer et al. 2023), caspase 1 (NM_012762.3) forward (5′‐ATG GAT TGC TGG ATG AAC T‐3′) and reverse (5′‐GAT AAC CTT GGG CTT GTC TT‐ 3′), and GAPDH (NM_017008.4) forward (5′‐TCC ATG ACA ACT TTG GCA TC‐3′) and reverse (5′‐CAT GTC AGA TCC ACC ACG GA‐3′) (Du et al. 2018). Livak method (2^−ΔΔCt^) was used to calculate expression (Heid et al. 1996).

#### ELISA for the Determination of caspase-1 Level

A rat caspase 1 ELISA kit (MyBioSource, California, USA) was used to determine caspase-1 concentration in the brains of the studied rat groups according to the manufacturer’s instructions. A microplate reader (Thermo Fisher Scientific, Waltham, USA) was used to measure the color intensity of the final product at 450 nm.

#### Histopathological Examination

The brain tissues were fixed in freshly prepared 10% formalin for histopathological evaluation and then embedded in paraffin to form paraffin blocks. Using Leica RM2135 microtome (Leica®, Berlin, Germany), the blocks were serially sliced (5-µm thickness) and mounted on glass slides, then stained with hematoxylin and eosin solution. After that, the brain sections were examined under a light microscope (CX43; Olympus, Tokyo, Japan) (Cardiff et al. 2014). A semiquantitative scoring was applied to evaluate neuronal shrinkage, glial cell apoptosis, neurophagia, and vacuolation to show the difference between studied groups. 0: absent, 1: mild, 2: moderate, 3: severe.

#### Immunohistochemical Staining of IL-1β

Brain sections were sliced from the paraffin blocks and mounted over polylysine slides. Then, using xylene, the sections were dewaxed, dehydrated by ethanol, and rehydrated using running tap water. Finally, the sections were prepared for antigen retrieval and stained with anti-IL-1β (1:200) (Pei et al. 2024). All the steps were performed according to the manufacturer protocol (Sigma Aldrich, St. Louis, USA). Microscopic images were examined to quantify the intensity of staining using Image J software (National Institutes of Health, Bethesda, USA). For semiquantitative immunohistochemistry, the number of positive cells was counted by an investigator blinded to the experimental groups. Under the microscope, the counting area for the glial cells of the cerebral cortex and the neurons of the pyramidal layer of the hippocampus area of each section was exactly the size of a complete field at ×400 magnification. Only the positive cells inside the glial cells of the cerebral cortex and pyramidal layer were counted. An average of six fields per immunostained slide was taken from each rat. Six rats were taken from each group.

#### Statistical Analysis

All data were expressed as mean ± SD or SE, using SPSS version 22 software for the statistical analysis. The one-way analysis of variance (ANOVA) method was used for statistical comparison between studied groups, followed by a post hoc Fisher’s least significant difference (LSD). Data with *p* < 0.05 were considered statistically significant.

## Results

### Phytochemical Analysis of CE

**Fig. 1:**
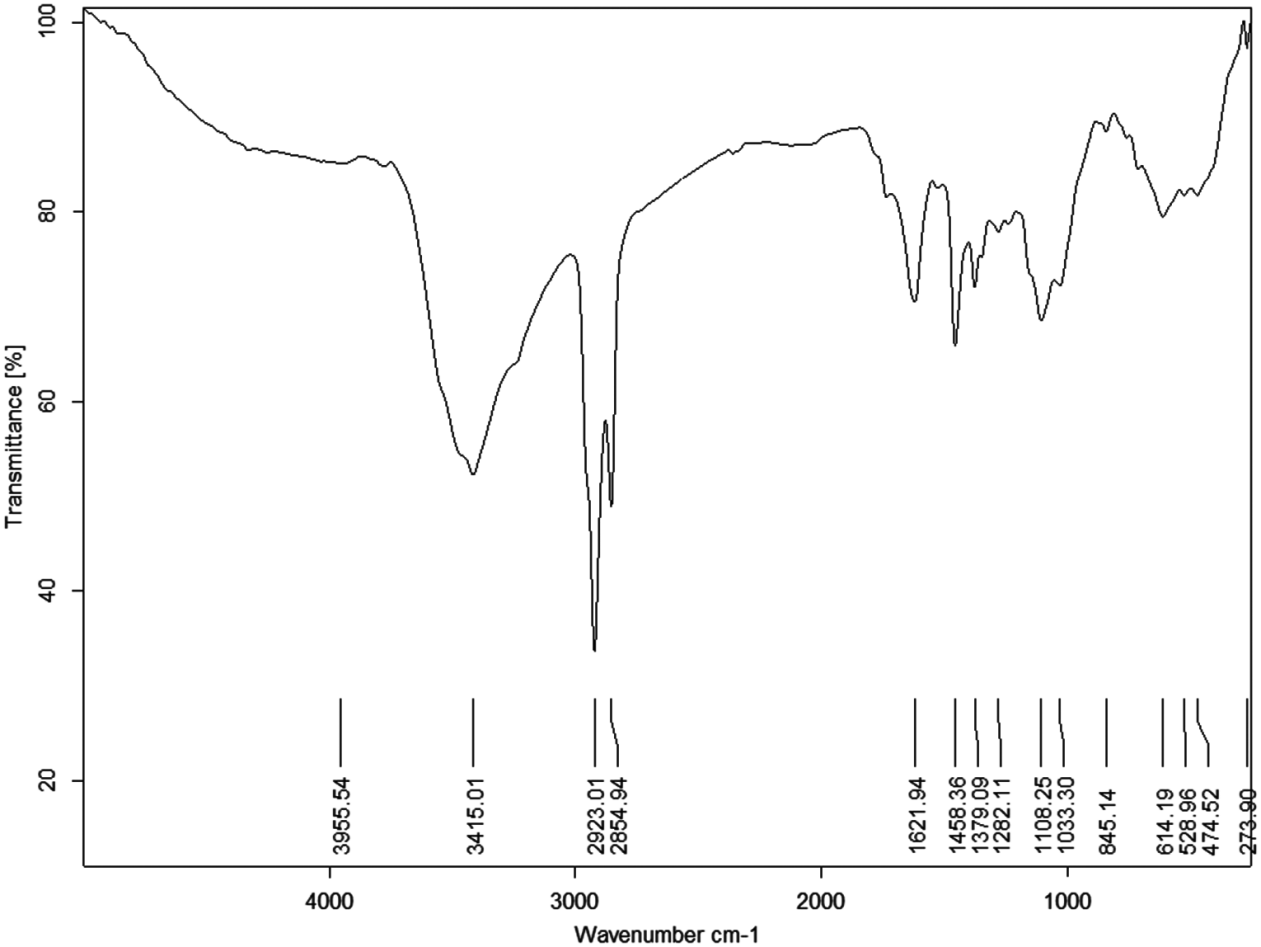
IR spectrum of the cranberry extract

The FT-IR fingerprint spectrum of the CE (Fig. 1) showed characteristic bands at 3415.01, 2923.01, 2854.94, 1621.94, 145,836, 1379.09, 1282.11, 1108.25, 1033.30, and 614.19 cm^-1^.

**Fig. 2:**
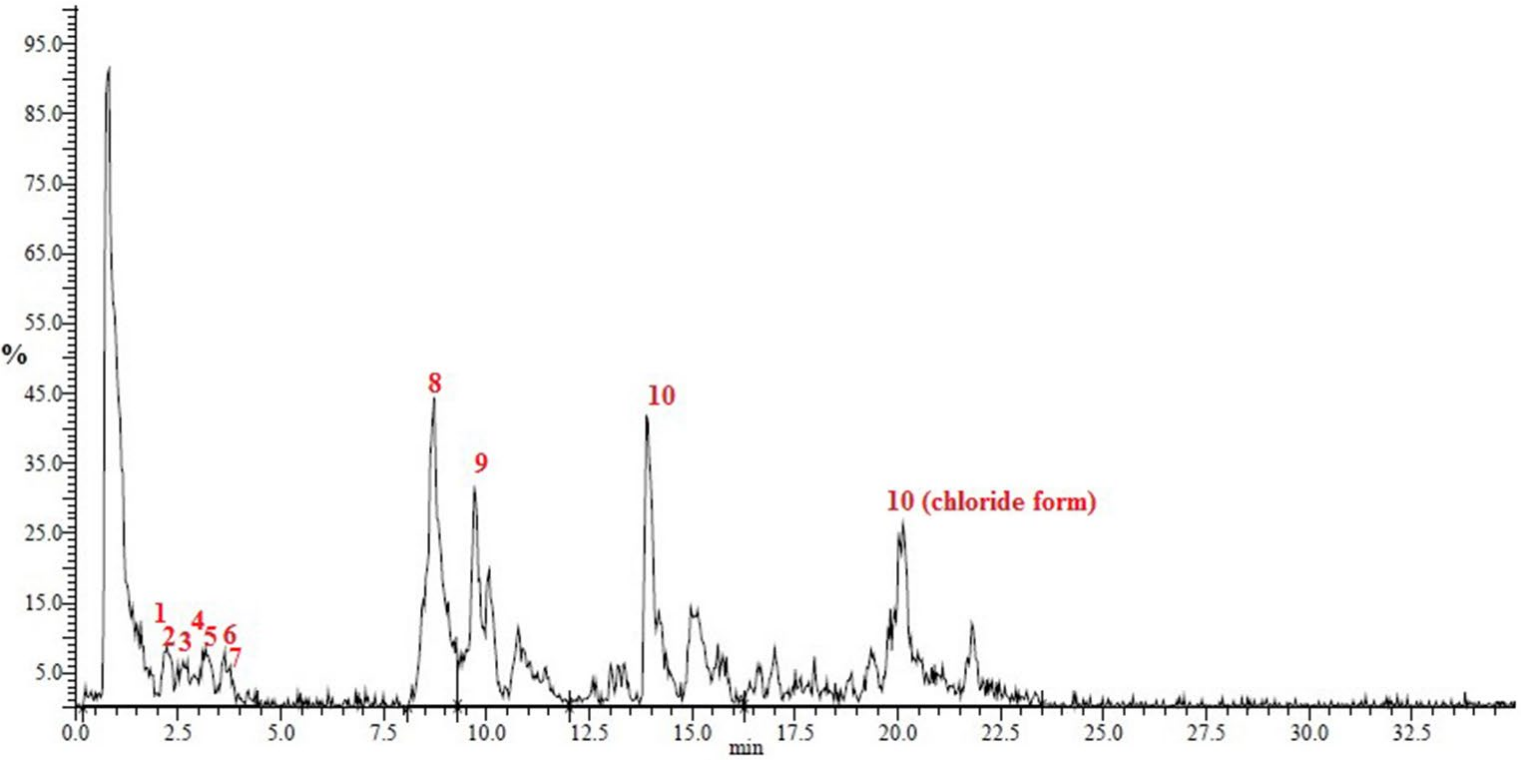
UPLC-ESI-MS total ion chromatogram of the cranberry extract in negative ion mode. Compounds identified are labelled in red (1–10)

The UPLC-ESI-MS/MS analysis conducted in negative ion mode indicated that the CE contained polyphenolic compounds (Fig. 2; Table 1). Cranberry contains tannins of the condensed type, which were identified in different varieties ^(16–18)^. The condensed tannins detected in our study in the CE were proanthocyanidins dimer and tetramer with pseudomolecular ions [M-H]^−^ at m/z 575 and 1169, respectively. These compounds exhibited MS/MS fragments at m/z 881 and 449, respectively. The anthocyanin detected was identified as cyanidin arabinoside at m/z 419 for the cation form and at m/z 455 for the chloride form, which showed MS/MS fragment at m/z 287 for the cyanidin part (Rajbhandari et al. 2011). The flavonol aglycones detected are quercetin and isorhamnetin with pseudomolecular ions [M-H]^−^ at m/z 301 and 315, respectively. The MS/MS fragmentation of the ion at m/z 301 yielded fragments at m/z 151 and 107, which are identical to quercetin MS/MS fragmentation in negative ion mode(Fabre et al. 2001; McNab et al. 2009; Yang et al. 2016). The MS/MS fragmentation of the ion at m/z 315 yielded fragments at m/z 300, 283, 271, and 151, which is consistent with isorhamnetin(McNab et al. 2009). In some cranberry varieties, quercetin is a major constituent and a marker for identification (Gudžinskaitė et al. 2020). Also, isorhamnetin is one of the flavonols identified in different cranberry varieties(Gudžinskaitė et al. 2020). Quercetin glycosides were previously detected in the cranberry extracts (Li et al. 2024; Rajbhandari et al. 2011; Gudžinskaitė et al. 2020). In our study, quercetin mono and dihexoside were detected. Additionally, myricetin hexoside was detected as an [M-H]^−^ at m/z 479, which, by the loss of the hexoside part (-162 Da), yielded m/z 317 for the aglycone myricetin.

**Table 1:** The UPLC-ESI-MS/MS analysis of the CE in negative ion mode

NO.	Rt min	[M-H]^−^ m/z	MS^2^ Ions m/z	Identification
1	2.25	1169	881	Proanthocyanidin tetramer
2	2.40	575	449	Proanthocyanidin dimer
3	2.85	625	301, 271,255	Quercetin dihexoside
4	3.15	479	317	Myricetin hexoside
5	3.25	463	301, 271,255	Quercetin hexoside
6	3.50	433	301,271,255	Quercetin pentoside
7	3.65	447	301, 271, 255	Quercetin rhamnoside
8	8.72	301	179, 151, 107	Quercetin
9	9.80	315	300, 283, 271, 151	Isorhamnetin
10	14.21 20.22	449 455	287	Cyanidin hexoside

### Biological Evaluation

#### Induction of Diabetes by Streptozotocin Injection Caused a Significant Increase in the Blood Glucose Level and a Significant Decrease in the Rat’s Total Body Weight

**Fig. 3:**
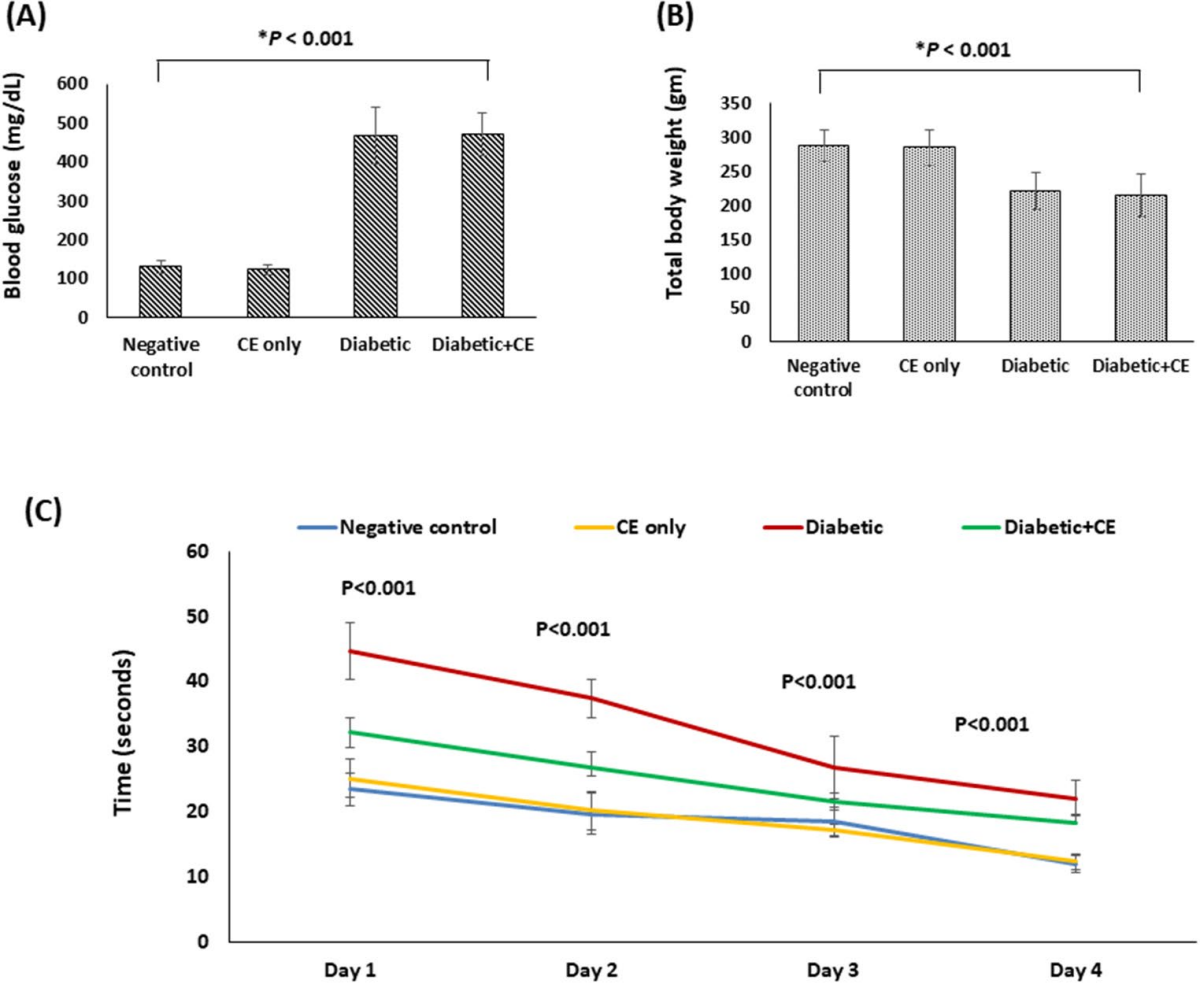
Effect of the cranberry extract (CE) prophylactic treatment on (**A**) Random blood glucose level, (**B**) Total body weight, and (**C**) The mean escape latencies of the studied rat groups. Data are revealed as mean ± SD, *n* = 6. Negative control (NC): non-diabetic rats taking the vehicle, CE only: non-diabetic rats treated with the CE 250 mg/kg/day orally for eight weeks, positive control (PC): diabetic rats taking the vehicle, and diabetic + CE: diabetic rats treated with the CE 250 mg/kg/day orally for eight weeks. Type 1 diabetes was induced in the rats by intraperitoneal injection of a single dose of 60 mg/kg streptozotocin

At the end of our experiment, random blood glucose and the total body weight of the rats in the studied groups were determined. Figures (3 A and B) showed that the induction of type 1 diabetes by intraperitoneal streptozotocin injection caused a significant increase in the random blood glucose level (466.50 ± 71.99 mg/dL, *p* < 0.001) and a significant decrease in the total body weight (220.67 ± 27.98 g, *p* < 0.001) of the rats in the positive control group compared to the normal rats in the negative control group (130.33 ± 17.13 mg/dL and 288.33 ± 22.94 g, respectively).

On the other hand, diabetic rats treated with the CE showed non significant difference in the random blood glucose level (471.83 ± 54.10 mg/dL) or the total body weight (213.83 ± 31.30 g) from the positive control group. Moreover, normal rats treated with the CE showed non significant difference in the random blood glucose level (122.67 ± 13.14 mg/dL) or the total body weight (284.83 ± 25.45 mg) from the rats in the negative control group Figures (3 A and B).

#### Effect of the CE on the Development of Diabetes-Induced Memory Deficit and Cognitive Impairment

The MWM test was carried out to evaluate the rats’ cognitive functions and memory retention. Diabetic rats in the positive control group took a long time to find the hidden platform compared to the rats in the negative control group (*p* < 0.001) (Fig. 3C). However, our data showed that the prophylactic treatment of the diabetic rats with the CE significantly improved the memory deficit and cognitive impairment induced by diabetes, as treated diabetic rats took a shorter time to find the hidden platform than rats in the positive control group (*p* < 0.005). Moreover, the time taken by the rats in the CE-only treated group to find the hidden platform showed non significant difference from those in the negative control group (Fig. 3C).

#### Effect of the CE on the Levels of MDA, GSH, and SOD Activity in the Brain of the Studied Rat Groups

**Fig. 4:**
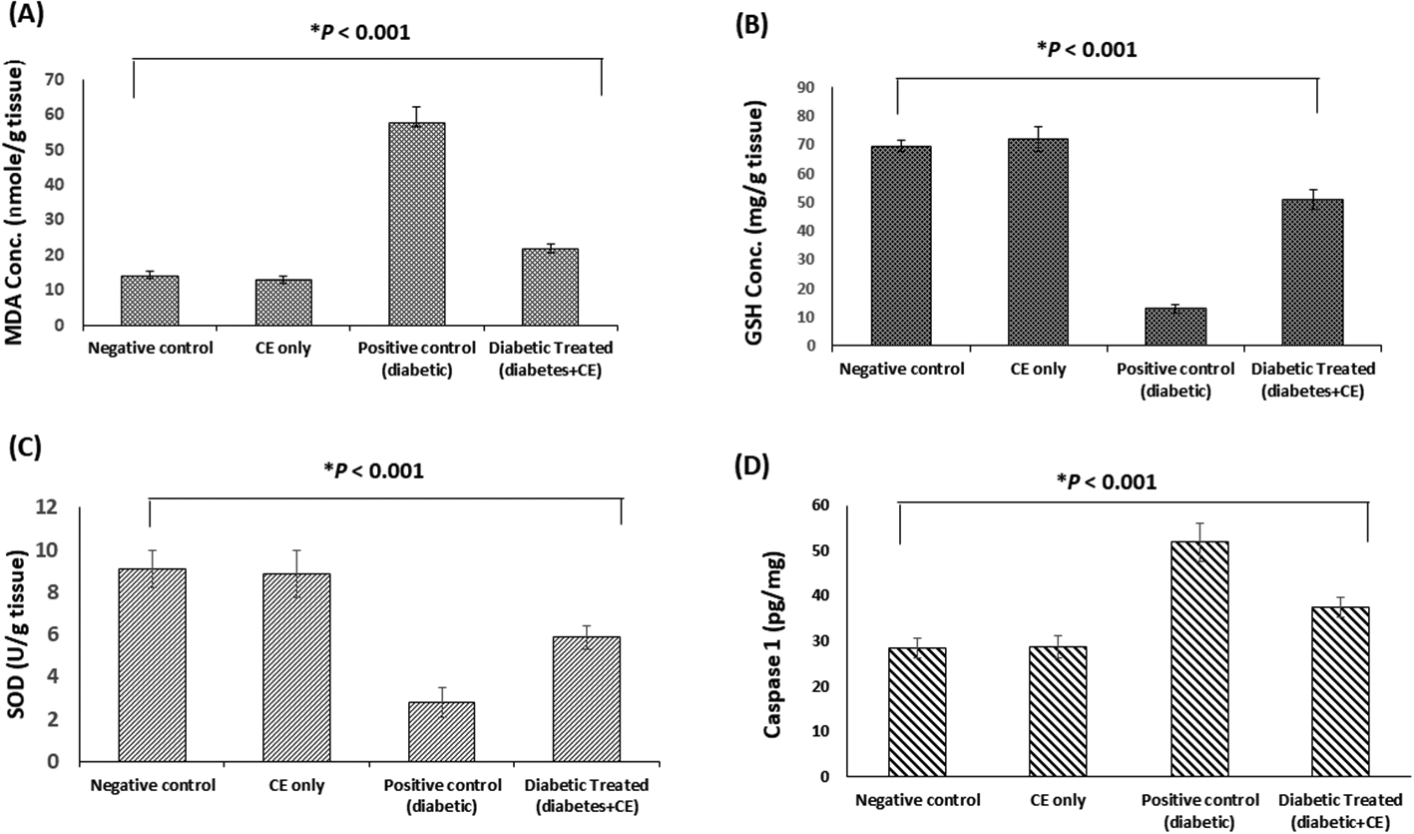
Effect of the cranberry extract (CE) prophylactic treatment on (**A**) Malondialdehyde (MDA) level, (**B**) Glutathione (GSH) level, and (**C**) Superoxide dismutase (SOD) activity, (**D**) Caspase 1 level in the brain of rats in the studied groups. Data are revealed as mean ± SD, *n* = 6. Negative control (NC): non-diabetic rats taking the vehicle, CE only: non-diabetic rats treated with the CE 250 mg/kg/day orally for eight weeks, positive control (PC): diabetic rats taking the vehicle, and diabetic treated: diabetic rats treated with the CE 250 mg/kg/day orally for eight weeks. Type 1 diabetes was induced in the rats by intraperitoneal injection of a single dose of 60 mg/kg streptozotocin

MDA level was measured in the brain tissue as an indicator of lipid peroxidation and oxidative stress. The brain tissue of the diabetic rats showed a significant increase (*p* < 0.005) in MDA levels (4.08-fold increase) compared to the healthy rats in the negative control group. On the other hand, the diabetic rats treated with the CE exhibited a significant decrease (*p* < 0.005) in the brain MDA values relative to the diabetic rats in the positive control group (62.5% decrease) (Fig. 4A).

On the other hand, the GSH level was measured as an antioxidant marker in the brain tissue of the rats. The positive control group revealed a significant decrease in the GSH level compared to the negative control group (81.6% decrease, *p* < 0.005). On the contrary, the diabetic group treated with the CE exhibited a marked increase in the GSH levels compared to the positive control group (3.9-fold increase, *p* < 0.005). The CE-only treated group showed non significant change in the MDA or GSH levels relative to the negative control group (Fig. 4B).

The activity of SOD was measured in the brain tissue as an antioxidant defense mechanism against oxidative stress. As illustrated in Figure (4 C), the brain tissue from the diabetic rats showed a significant decrease in the SOD activity (*p* < 0.005, 69.3% decrease) compared to the negative control rats. On the other hand, treating the diabetic rats with the CE significantly improved the SOD activity relative to the positive control group (2.15-fold increase, *p* < 0.005). The CE-only treated rats showed that the SOD activity was near normal.

#### Effect of CE on Caspase 1 Level in the Brain of the Studied Rat Groups

The concentration of caspase-1 (a marker of pyroptosis) was determined in the brains of the studied rat groups. The concentration of caspase 1 was significantly higher (*p* < 0.001) in the brain of untreated diabetic rats (51.82 ± 4.15 pg/mg) compared to its concentration in the brain of normal rats (28.43 ± 2.26 pg/mg). Prophylactic administration of PPE to the diabetic rats significantly decreased (*p* < 0.001) caspase 1 concentration in the brain (37.50 ± 2.14 pg/mg) compared to the untreated diabetic rats. Moreover, the concentration of caspase 1 in the brain of the normal rats treated with the PPE (28.63 ± 2.52 pg/mg) showed non significant difference from its concentration in the brain of normal rats in the negative control group (Fig. 4D).

#### Effect of the CE on the Gene Expression of GDNF, lncRNA GAS-5, and Pyroptosis Genes in the Brain of the Studied Rat Groups

**Fig. 5:**
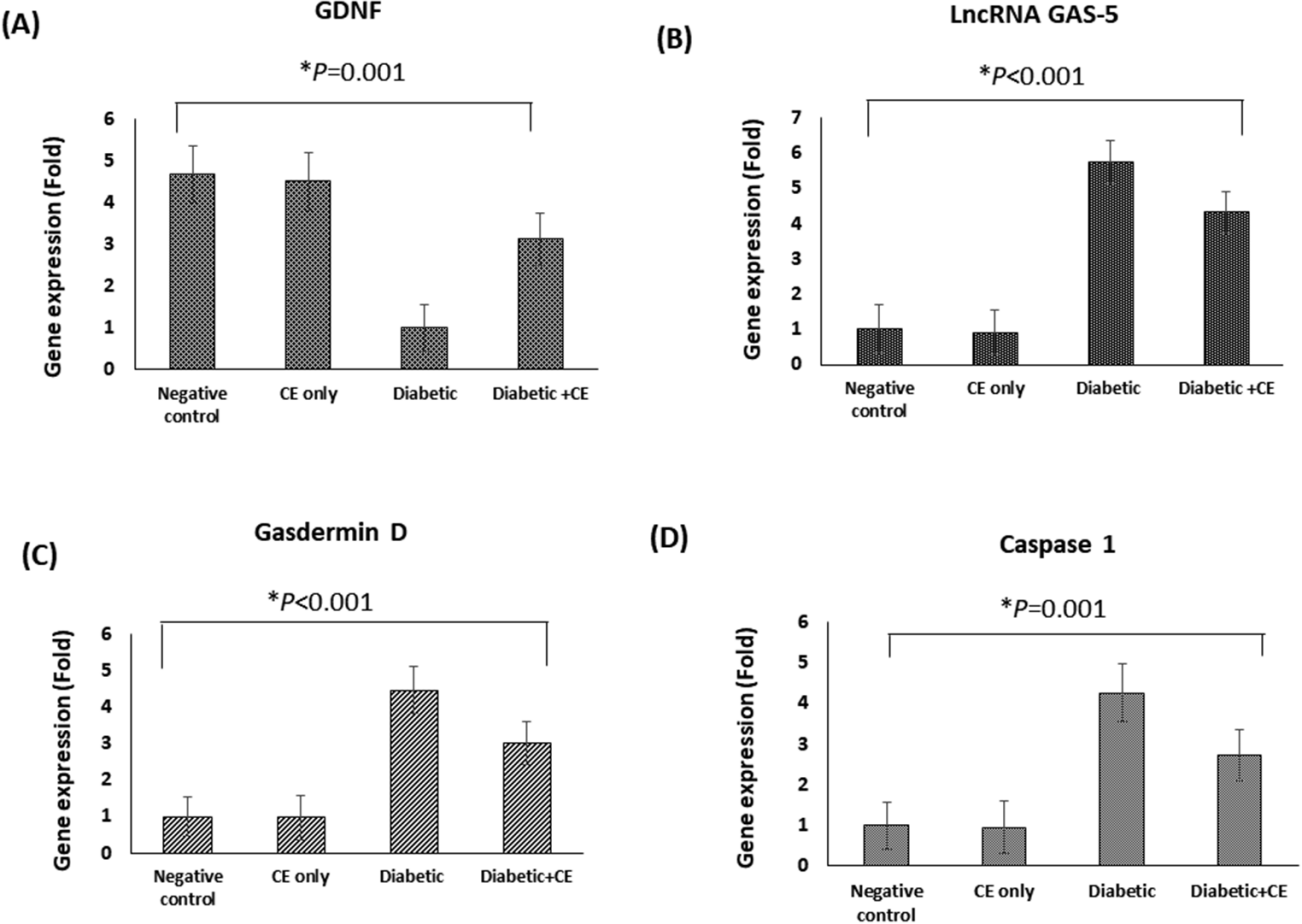
Fold gene expression analysis in the brain of the studied rat groups (**A**) Glial cell-derived neurotrophic factor (GDNF), (**B**) Long non-coding RNA growth-arrest-specific-5 (LncRNA GAS-5), (**C**) Gasdermin D, and (**D**) Caspase 1. Data are revealed as mean ± SD, *n* = 3. Negative control (NC): non-diabetic rats taking the vehicle, CE only: non-diabetic rats treated with the cranberry extract 250 mg/kg/day orally for eight weeks, positive control (PC): diabetic rats taking the vehicle, and diabetic + CE: diabetic rats treated with the cranberry extract 250 mg/kg/day orally for eight weeks. Type 1 diabetes was induced in the rats by intraperitoneal injection of a single dose of 60 mg/kg streptozotocin

In the present study, real-time PCR was used to determine the gene expression of GDNF in the brains of rats as a neuroprotective marker. The current real-time PCR data demonstrated that the gene expression of GDNF in the brain of the diabetic rats in the positive control group (1 ± 0.57 fold) was significantly lower than its gene expression in the brain of the normal rats in the negative control group (4.68 ± 0.67-fold, *p* < 0.005). Meanwhile, the prophylactic treatment with the CE significantly increased the gene expression of GDNF in the brains of diabetic rats (3.12 ± 0.62-fold, *p* < 0.05). Our data showed that there was non significant difference between the gene expression of GDNF in the brain of the CE-only treated group (4.51 ± 0.69 fold) and the brain of the rats in the negative control group (4.68 ± 0.67 fold) (Figure. 5 A).

Moreover, our real-time PCR data demonstrated that the levels of the gene expression of lncRNA-GAS-5 and markers related to pyroptosis (gasdermin D and caspase 1) in the brain of the diabetic rats in the positive control group (5.74 ± 0.61, 4.47 ± 0.65, and 4.25 ± 0.71-fold, respectively) were significantly higher than their levels in the brain of the normal rats in the negative control group (1-fold, *p* < 0.005). In addition, the prophylactic treatment with the CE significantly decreased the gene expression of lncRNA-GAS-5 and pyroptosis markers (gasdermin D and caspase 1) in the brain of the diabetic rats (4.32 ± 0.60, 3.02 ± 0.58, and 2.73 ± 0.62 fold, respectively, *p* < 0.05). Our data showed that the levels of the gene expression of lncRNA-GAS-5 and pyroptosis markers (gasdermin D and caspase 1) in the brain of the CE-only treated group (0.91 ± 0.64, 0.97 ± 0.62, and 0.95 ± 0.65-fold, respectively) showed non significant difference from their levels in the brain of the rats in the negative control group (1-fold) (Figs. 5B, C, and D).

#### Effect of the CE on Diabetes-Induced Histopathological Alterations in the Brain

**Fig. 6:**
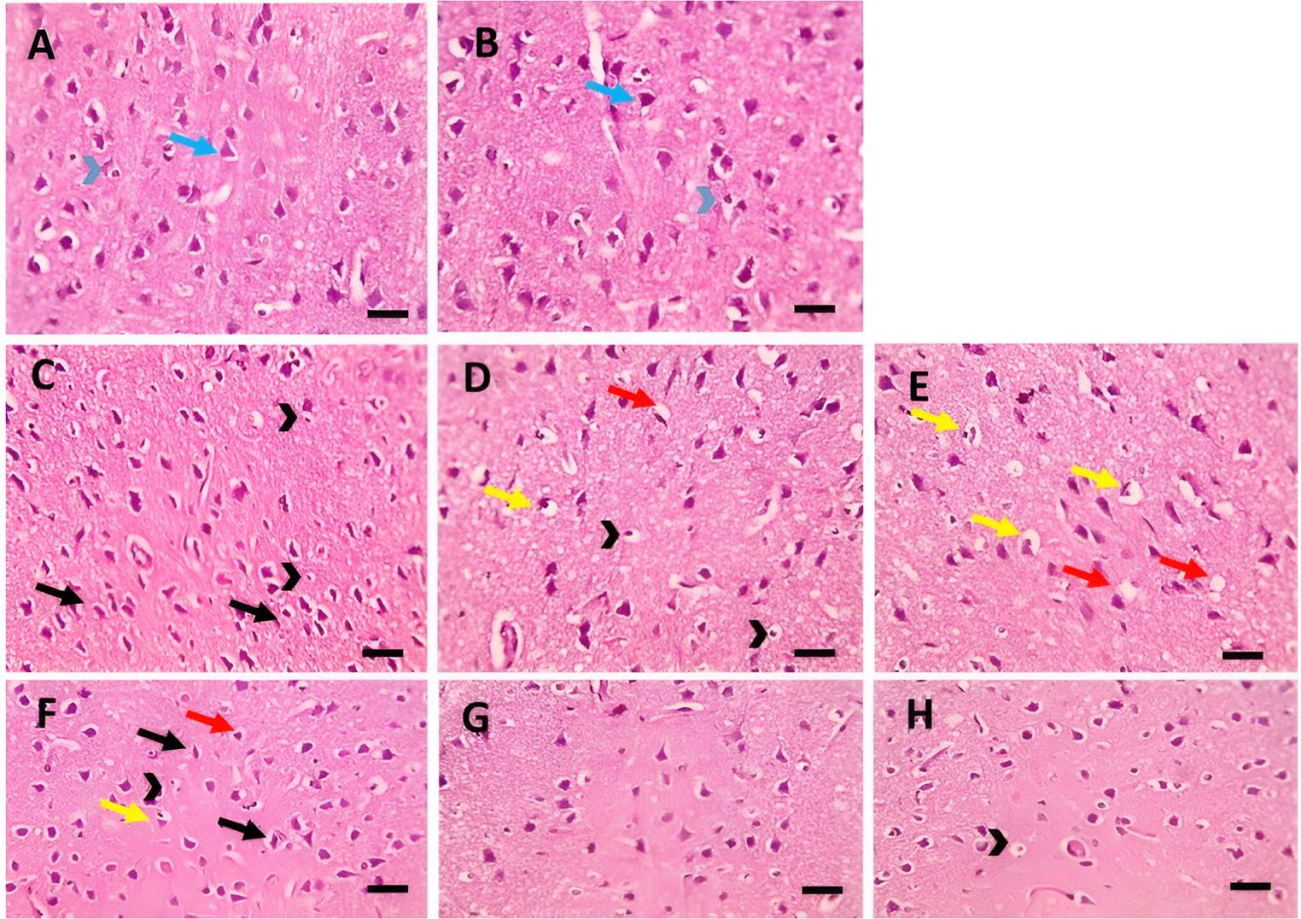
**Microscopic pictures of H&E-stained cerebral cortex of the studied rat’s groups.** (**A** and **B**): Cerebral cortical sections from the negative control group and cranberry extract-only treated group, respectively, showing normal neurons (blue arrows), glial cells (blue arrowheads) and neuropil. (**C** -**E**): Cerebral cortical sections from the positive control group (diabetic rats) showed marked shrinkage and degeneration of many neurons (black arrows), many apoptotic glial cells (black arrowheads), prominent neurophagia (yellow arrows) and vacuolation in neuropil (red arrows). (**F**-**H**): Cerebral cortical section from diabetic rats treated with cranberry extract showing decreased numbers of affected neurons (black arrows) and glial cells (black arrowheads), mild neurophagia (yellow arrows) and mild vacuolation in neuropil (red arrows). magnification X: 400 bar 50 μm

In the present study, the microscopic examination of the H&E-stained sections of the control group and the normal rats’ group treated with the CE revealed the normal histological structure of the cerebral cortical tissue, where a mixture of medium-to-large-sized neurons and glial cells were depicted in a fibrillary background (neuropil), (Fig. 6A and B, respectively). In the diabetic group, neuron degeneration was characterized by cell body shrinkage and intensely stained eosinophilic cytoplasm. Some neurons showed normal-appearing nuclei, whereas others have pyknotic or fragmented nuclei. Additionally, frequent apoptotic glial cells and finely vacuolated neuropils were noted (Fig. 6C and D). In the diabetic group pre-treated with CE, the number of degenerated neurons and apoptotic glial cells remarkably decreased compared to the diabetic group (Fig. 6E).

Semiquantitative scoring was used to evaluate the neuronal shrinkage, glial cell apoptosis, neurophagia, and vacuolation in the cerebral cortex of the studied rat’s groups in brain sections. In general, the scores in each group were consistent. Both negative control and non-diabetic rats treated with CE were given a score of (0) regarding neuronal shrinkage, glial cell apoptosis, neurophagia, and vacuolation. The positive control group (diabetic rats) showed significant increase in neuronal shrinkage, glial cell apoptosis, neurophagia, and vacuolation scoring versus negative control group. Interestingly, diabetic rats pre-treated with CE showed significant decrease in scoring regarding neuronal shrinkage, glial cell apoptosis, neurophagia, and vacuolatio when compared to positive control group (Fig. 6; Table 2).

**Table 2:** Semiquantitative scoring of neuronal shrinkage, glial cell apoptosis, neurophagia, and vacuolation in the cerebral cortex of the studied groups

	Negative control	Positive control (Diabetic rats)	Diabetic rats pre-treated with CE	Non-diabetic rats treated with CE
Neuronal shrinkage	0.0 ± 0.0	2.5 ± 0.15^a^	0.5 ± 0.15^b^	0.0 ± 0.0
Glial cell apoptosis	0.0 ± 0.0	2.5 ± 0.15^a^	0.6 ± 0.14^b^	0.0 ± 0.0
Neurophagia	0.0 ± 0.0	2.5 ± 0.15^a^	0.8 ± 0.2^b^	0.0 ± 0.0
Vacuolation	0.0 ± 0.0	1.6 ± 0.14^a^	0.5 ± 0.15^b^	0.0 ± 0.0

Data are expressed as mean ± SD (*n* = 12). Significance was set at *p* < 0.05, a: significant vs. negative control group, b: significant vs. positive control group (diabetic rats). CE: cranberry extract. 0: absent, 1: mild, 2: moderate, 3: severe

**Fig. 7:**
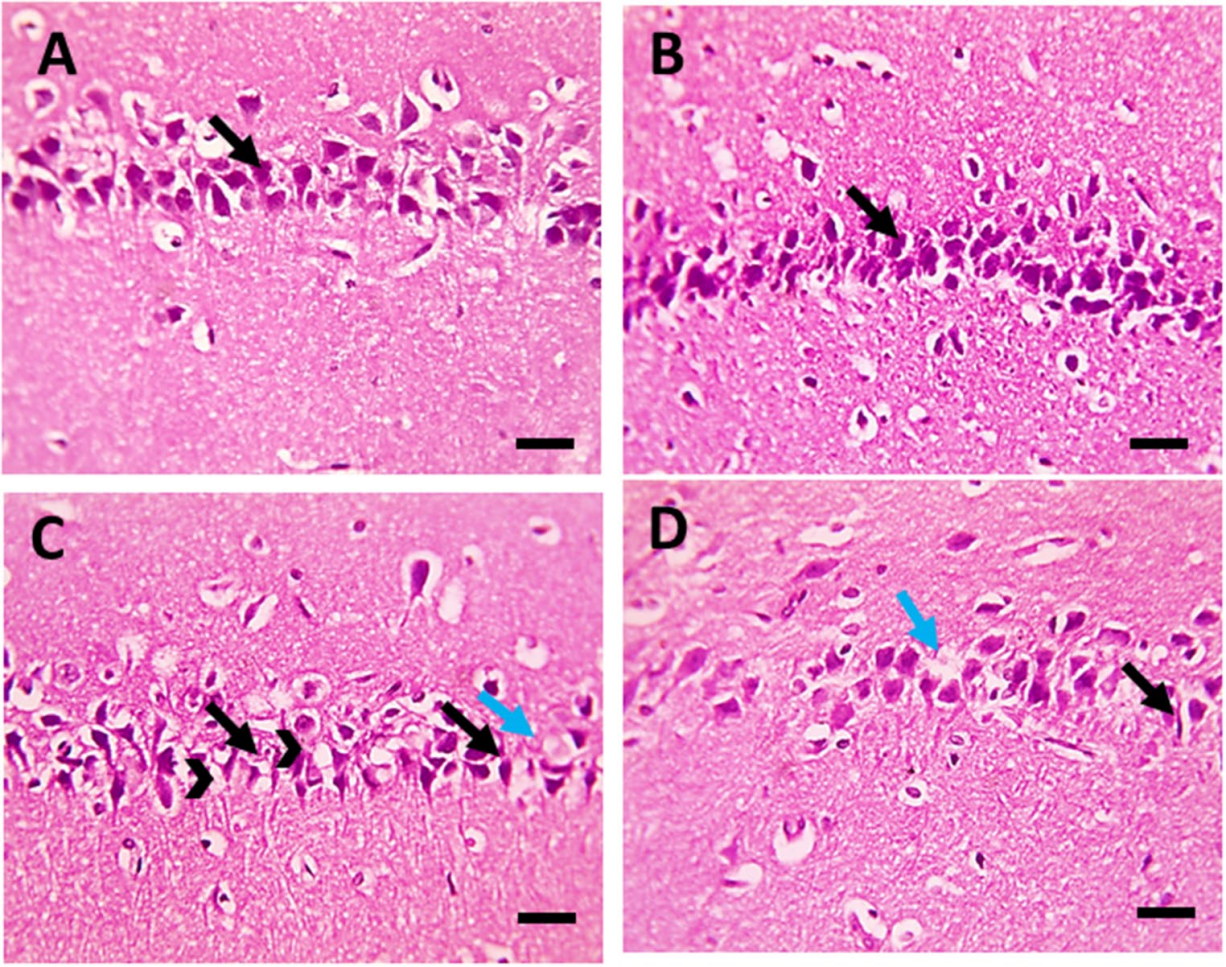
Microscopic pictures of H&E-stained hippocampal sections showing: **A**, **B**) Normal neurons (black arrows) in the pyramidal layer in the control group and normal rats treated with cranberry. **C**) Marked shrinkage & degeneration of many neurons (black arrows), few apoptotic neurons (blue arrows) with many vacuolations (black arrowheads) in the diabetic group. **D**) Few degenerated neurons (black arrow) and few apoptotic neurons (blue arrows) in the diabetic group pre-treated with cranberry. Magnification: x400, scale bar 50 μm

Microscopic examination of the H&E-stained hippocampal sections revealed normal distribution and histology of neurons in the pyramidal cell layer in the control and the normal rats treated with CE groups (Fig. 7A and B, respectively). Marked neurodegeneration was noted in the hippocampus of diabetic rats, as well as a few apoptotic neurons. In addition, a vacuolar alteration was seen within the cytoplasm of neurons (Fig. 7C). Pre-treatment with CE in diabetic rats ameliorated the neuron loss; only a few degenerated and apoptotic neurons were observed (Fig. 7D).

#### Effect of the CE on IL-1β

**Fig. 8:**
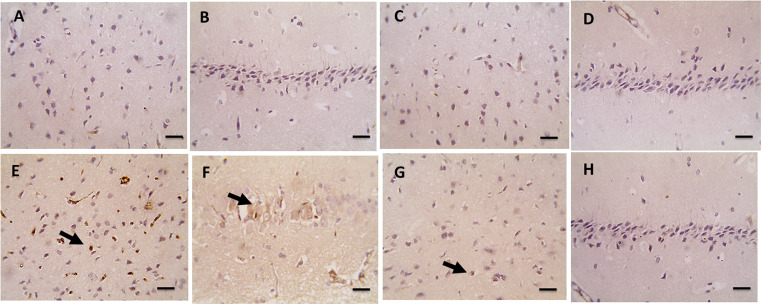
Microscopic pictures of immunostained brain sections against IL-1β in the studied rat groups. (**A** and **B**): Brain sections from rats in the negative control group showed negative staining in neurons and glial cells in the examined cerebral cortex and hippocampal pyramidal layer, respectively. (**C** and **D**): Brain sections from non-diabetic rats treated with cranberry extract showed negative staining in neurons and glial cells in the examined cerebral cortex and hippocampal pyramidal layer, respectively. (**E** and **F**): Brain sections from diabetic rats showing marked positive staining in glial cells (arrows) in the cerebral cortex and neurons (arrows) of the hippocampal pyramidal layer, respectively. (**G** and **H**): Brain sections from diabetic rats treated with cranberry extract showed weak positive staining in a few glial cells and a few cerebral cortical sections (arrows) and negative staining in neurons of the hippocampal pyramidal layer, respectively. Magnification: x400, scale bar 50 μm

Immunostaining of the pro-inflammatory cytokine IL-1β (a marker of pyroptosis) in the brain tissue was performed. Interpretation of the immunohistochemically stained slides against IL-1β revealed no immunoreactive glial cells or neurons in the cerebral cortex and the hippocampal pyramidal layer, respectively, of the control group (Fig. 8A and B, respectively) and the normal rats’ group treated with the cranberry (Fig. 8C and D, respectively). However, IL-1β immunoreactivity was intense in the cerebral cortex glial cells and the hippocampal pyramidal layer neurons in the diabetic rats from the positive control group (Fig. 8E and F, respectively). Notably, the IL-1β immunoreactivity declined to weak positivity, as noted in scarce glial cells in a few cerebral cortical sections and negative staining in neurons of the hippocampal pyramidal layer in the diabetic rats’ group treated with CE (Fig. 8G and H, respectively).

Semiquantitative scoring was used to evaluate the expression of IL-1β in brain sections. An average of six fields per immunostained slide was taken from each rat. Six rats were taken from each group. In general, the scores in each group were consistent. Both negative control and non-diabetic rats treated with CE were given a score of (0), denoting negative immunostaining. In the positive control group (diabetic rats), IL-1β positive immunostaining was noted in 40 to 50% of the cerebral cortex glial cells and 30 to 55% of neurons in the pyramidal layer of the hippocampus. Interestingly, immunopositivity was only detected in 5% of the glial cells of the cerebral cortex of two rats and 5% of hippocampal neurons in the pyramidal layer of only one rat (Table 3).

**Table 3:** Semiquantitative scoring to evaluate the expression of Il-1β in the brain cortex and hippocampus

	Negative control (%)	Positive control (Diabetic rats) (%)	Diabetic rats pre-treated with CE (%)	Non-diabetic rats treated with CE (%)
Cortex	0 0 0 0 0 0	40 40 50 50 50 45	5 0 0 0 5 0	0 0 0 0 0 0
Hippocampus	0 0 0 0 0 0	30 40 40 45 55 40	0 5 0 0 0 0	0 0 0 0 0 0

The given scores denote the percentage of the Il-1β immunostained cells in the glial cells of the cerebral cortex and the neurons of the pyramidal layer of the hippocampus in the four tested groups of rats: negative control, positive control (diabetic rats), diabetic rats pre-treated with CE and non-diabetic rats treated with CE

## Discussion

Cognitive dysfunction or dementia is a serious complication and highly prevalent comorbidity of uncontrolled diabetes (Biessels and Whitmer 2020; Yang et al. 2022). This study aimed to investigate the protective effect of the CE against diabetes-induced brain injury and cognitive impairment in rats and to determine the possible underlying molecular mechanism.

The phytochemical analysis of the CE extract used in our study revealed that anthocyanins, namely cyanidin-3-*O*-arabinoside, quercetin (aglycone and glycosides) compounds, and procyanidins are the main components. Anthocyanins are known for their protective effects on neurodegenerative diseases(Afzal et al. 2019; H et al. 2023). Additionally, quercetin can prevent neurodegeneration-associated memory loss, as discussed by Jakaria et al. (Jakaria et al. 2019). Proanthocyanidins exhibit neuroprotection effects and can improve memory state (Zhao et al. 2019). These components can account for the observed results in our study.

In the present study, the MWM test was performed to evaluate the rats’ cognitive functions and their memory retention (Yadav et al. 2017). MWM test is a common behavioral test that measures the spatial learning for rodents. This test has proven to be a unique robust and reliable tool which is strongly correlated with hippocampal synaptic plasticity and N-methyl-D-aspartate (NMDA) receptor function. The NMDA receptor is the receptor of glutamate which is considered the main excitatory neurotransmitter in the brain and plays an essential role in synaptic plasticity, a special neuronal mechanism that believed to be the cornerstone of memory formation (Yadav et al. 2017; Vorhees and Williams 2006).

Our data from the MWM test showed that the prophylactic treatment with the CE protects against the development of diabetes-induced memory deficit and cognitive impairment, evidenced by a significant decrease in the escape latency time. The current results were in line with Guo et al. (Guo et al. 2016), who found that supplementation of the CE protects against the development of memory deficit in the Alzheimer’s model *via* reducing the Aβ toxicity.

The high blood glucose level in diabetes contributes to the production of ROS. High oxidative stress is strongly correlated with brain injury and cognitive impairment(Chen et al. 2018; Rom et al. 2019). The current study revealed that diabetes induces a significant decrease (*p* < 0.005) in the cytoprotective GSH and SOD activity in the brain tissue. In addition, the lipid peroxidation marker (MDA) was significantly increased (*p* < 0.005) in the brain tissue by diabetes induction. More interestingly, the prophylactic treatment with the CE significantly increased the level of GSH and SOD activity as well as reduced lipid peroxidation in the brain of diabetic rats (*p* < 0.005). The beneficial use of proanthocyanidins for glycemic control in diabetics was reported in a previous study where it showed anti-diabetic action by significantly decreasing blood glucose level and increasing insulin level in addition to improving lipid profile (El-Ashmawy et al. 2022). In line with our results, procyanidin has sucssessfully, boosted SOD activity and diminished MDA content in neuroblastoma cells (Luo et al. 2018). Likewise, quercetin hexoside was shown to suppress the acrylamide-induced oxidative damage through inhibiting the mitochondrial membrane lipid peroxidation and glutathione depletion (Zhang et al. 2017).

GDNF is a member of the transforming growth factor family, which has a neuroprotective effect against various injuries (Tenenbaum and Humbert-Claude 2017). Moreover, it was reported that the upregulation of GDNF attenuates the induced learning and memory deficit in rats by inhibiting neuroinflammation(Torika et al. 2018; Zhang et al. 2014). In the current study, the induction of diabetes significantly reduced the gene expression of the neuroprotective GDNF in the brain tissue of the rats in the positive control group. Meanwhile, the prophylactic treatment of diabetic rats with CE significantly increased the gene expression of the neuroprotective GDNF in the brain of diabetic rats. This effect could be explained by the ability of GDNF to increase Bcl-2 level which, in turn, has an inhibitory effect on the pyroptosis activation. In addition, it was reported previously that GDNF rescues hyperglycemia-induced diabetic enteric neuropathy through activation of the PI3K/Akt pathway. It has been suggested that inhibition of PI3K/p-AKT pathway triggered cell pyroptosis (Guo et al. 2016; Shi and Kehrl 2019; Anitha et al. 2006; Yu et al. 2022).

Pyroptosis is caspase-1-dependent inflammatory programmed cell death, which is provoked as a response to cellular damage, oxidative stress, or metabolic imbalances (Xue et al. 2019). Pyroptosis is initially mediated by forming an inflammasome complex that activates caspase 1. The activated caspase 1 converts the inactive proinflammatory cytokine IL-1β into its active form, which is released out of the cell by gasdermin D (Zheng and Li 2020).

The current study revealed that the prophylactic treatment with the CE significantly inhibited pyroptosis in the brain of the diabetic rats, which was demonstrated by a significant decrease in the gene expression of caspase 1 and gasdermin D as well as a marked reduction in the number of IL-1β immune-stained positive cells in the brain. Moreover, the protein level of caspase 1 (IL 1 converting enzyme) in the brain of diabetic rats treated with CE was significantly lower than its level in the brain of diabetic non-treated rats, supporting the data of gene expression.

The present findings were supported by a recent study by Liu et al. (Liu et al. 2022), who reported that the activation of pyroptosis in diabetes, possibly *via* high mobility group box 1, disrupts the blood-brain barrier, causing cognitive impairment and dementia. Interestingly, Ye et al. (Ye et al. 2018) revealed that the inhibition of pyroptosis by gastrodin has a neuroprotective effect and could improve diabetes-induced cognitive dysfunction. Supporting our findings, in an earlier study, quercetin and myricetin have shown to ameliorate dexamethasone-induced muscle atrophy by reducing caspase 1, gasdermin D as well as IL-1β (Park et al. 2023). Furthermore, Isorhamnetin was speculated to have cytoprotective potential by suppressing by NLRP3 and caspase 1, in a model of acetaminophen-induced liver injury (Rousta et al. 2022). Moreover, Wei et al. (2022) reported that quercetin could inhibit pyroptosis pathway in diabetic cardiomyopathy. Further, El-Shaer et al., (2023) revealed that quercetin has a potent protective effect on the pulmonary dysfunction in diabetic rats *via* the inhibition of NLRP3 signaling pathway(Scassellati et al. 2021; El-Shaer et al. 2023; Wei et al. 2022).

GAS-5 is a lncRNA essential in regulating various diseases, including diabetes (Shi et al. 2019). GAS-5 is correlated to NLRP3 activation and the progression of cognitive impairment in Parkinson’s disease (Xu et al. 2020). Our real-time PCR data showed that the gene expression level of lncRNA GAS-5 in the brain of the diabetic rats in the positive control group was significantly higher than in the brain of normal rats in the negative control group (by more than fivefold). More interestingly, prophylactic treatment of the diabetic rats with the CE significantly decreased the gene expression of lncRNA-GAS-5 in the brain (by more than fourfold). In a previous study, myrcetin– an active compound in CE– possessed an anti-inflammatory effect and was capable of ameliorating cell apoptosis and cell inflammation *via* regulation of GAS5 which in line with our study (Bai et al. 2021).

This study showed for the first time the neuroprotective effect of the CE on diabetes induced-brain injury and memory deficit, which could be due to its ability to inhibit pyroptosis *via* downregulation of lncRNA GAS-5.

The histopathological examination of the cerebral cortex and hippocampus sections of the brain of the studied rat groups supported our biochemical findings. The induction of diabetes in rats led to a change in the normal brain histological structure, which was demonstrated by injury and degradation of the neurons of the cerebral cortex and the hippocampus. On the other hand, the prophylactic administration of the CE to diabetic rats protected against the development of diabetes-induced histopathological alterations in the cerebral cortex and hippocampus of the brain.

Interestingly, Intranasal insulin, metformin, incretins, and thiazolidinediones are a few examples of anti-diabetic drugs that have been demonstrated in human studies to improve cognition and memory in individuals with mild cognitive impairment and Alzheimer’s disease (Michailidis et al. 2022). In a recent study by Flanagan et al., (Flanagan et al. 2022) the investigators revealed that over 12 weeks, daily cranberry supplementation, equivalent to a tiny cup of cranberries, improved episodic memory ability and brain functioning.

Dietary intervention has become a crucial part of management strategies for chronic illnesses, including diabetes. Therefore, it is necessary and justified to launch activities and campaigns to boost the consumption of fruits and vegetables. One effective method for preventing and treating diabetes-associated comorbidities is encouraging people to eat more fruits and vegetables (Flanagan et al. 2022). Specific research-based evidence may be included in counseling, such as encouraging diabetic patients to consume CE to avert cognitive impairment.

Nevertheless, our research provides valuable insights and paves the way for more comprehensive studies in the future aimed at further dissecting the effect of CE at a molecular level. The understanding of the effect of CE on pyroptosis signaling pathway require future mechanistic studies to demonstrate the correlation. Further detailed research could explore the specific mechanism of action of the main active ingredients of CE in against diabetes-induced cognitive dysfunction as well as the exact role of CE in the inhibition of NLRP3 inflammasome activation. A limitation of our study is the small sample size due to complications associated with diabetes progression. Further studies should consider larger number of animals in diabetic groups to account for potential losses and ensure robust data analysis.

## Conclusions

Due to its unique antioxidant and anti-inflammatory activity, the CE showed a protective effect against diabetes-induced cognitive dysfunction in rats. The protective effect of the CE on the diabetic brain could be due to the downregulation of lncRNA GAS-5 and subsequent inhibition of the pyroptosis pathway. The CE could be a promising therapy to protect against the development of diabetes-induced neurotoxicity and memory deficit. However, further pre-clinical and clinical research is recommended to investigate the effect of the pure natural extracted compounds that present in cranberry (cyanidin arabinoside, quercetin, isorhamnetin, and myricetin) on the treatment and prevention of cognitive impairment and the underlying molecular mechanisms. Moreover, the toxicity of long term use of cranberry extract should be studied.

## Data Availability

I do not have any research data outside the submitted manuscript file.

## Abbreviations

CE: Cranberry extract
ESI: Electrospray ionization
GAS-5: Growth arrest-specific 5
GDNF: Glial cell-derived neurotrophic factor
GSH: Glutathione
IL-1β: Interleukin-1beta
lncRNAs: Long non-coding RNAs
MDA: Malondialdehyde
MWM: Morris water maze
NLRP3: Nucleotide-binding domain-like receptor protein 3
NOD: Nucleotide-binding domain
ROS: Reactive oxygen species
SOD: Superoxide dismutase

## References

[CR42] Afzal M, Redha A, AlHasan R (2019) Anthocyanins potentially contribute to defense against Alzheimer’s disease. Mol Basel Switz 24:4255 10.3390/molecules24234255 PMC6930593 31766696

[CR29] Ahmadiantehrani S, Barak S, Ron D (2014) GDNF is a novel ethanol-responsive gene in the VTA: implications for the development and persistence of excessive drinking. Addict Biol 19:623 23298382 10.1111/adb.12028 PMC3626755

[CR55] Anitha M et al (2006) GDNF rescues hyperglycemia-induced diabetic enteric neuropathy through activation of the PI3K/Akt pathway. J Clin Invest 116:344–356 16453021 10.1172/JCI26295 PMC1359053

[CR23] Assar DH et al (2021a) Wound healing potential of licorice extract in rat model: antioxidants, histopathological, immunohistochemical and gene expression evidences. Biomed Pharmacother Biomedecine Pharmacother 143:112151 10.1016/j.biopha.2021.112151 34507115

[CR24] Assar DH et al (2021b) Ameliorative effects of Aspergillus Awamori against the initiation of hepatocarcinogenesis induced by diethylnitrosamine in a rat model: regulation of Cyp19 and p53 gene expression. Antioxid Basel Switz 10:922 10.3390/antiox10060922 PMC8228954 34200190

[CR26] Babu PS, Srinivasan K (1997) Influence of dietary capsaicin and onion on the metabolic abnormalities associated with streptozotocin induced diabetes mellitus. Mol Cell Biochem 175:49–57 9350033 10.1023/a:1006881027166

[CR61] Bai Y et al (2021) Myricetin ameliorates ox-LDL-induced HUVECs apoptosis and inflammation via LncRNA GAS5 upregulating the expression of miR-29a-3p. Sci Rep 11:19637 34608195 10.1038/s41598-021-98916-7 PMC8490408

[CR40] Biessels GJ, Whitmer RA (2020) Cognitive dysfunction in diabetes: how to implement emerging guidelines. Diabetologia 63:3–9 31420699 10.1007/s00125-019-04977-9 PMC6890615

[CR34] Cardiff RD, Miller CH, Munn RJ (2014) Manual hematoxylin and eosin staining of mouse tissue sections. *Cold Spring Harb. Protoc.* 655–658 (2014) 10.1101/pdb.prot073411 24890205

[CR7] Che H et al (2020) Melatonin exerts neuroprotective effects by inhibiting neuronal pyroptosis and autophagy in STZ-induced diabetic mice. FASEB J Off Publ Fed Am Soc Exp Biol 34:14042–14054 10.1096/fj.202001328R 32910484

[CR30] Chen D, Zhang M (2021) GAS5 regulates diabetic cardiomyopathy via miR–221–3p/p27 axis–associated autophagy. Mol Med Rep 23:135 33313941 10.3892/mmr.2020.11774 PMC7751493

[CR1] Chen R et al (2018) Morphological and pathological characteristics of brain in diabetic encephalopathy. J Alzheimers Dis JAD 65:15–28 30040723 10.3233/JAD-180314

[CR32] Du S-Q et al (2018) Acupuncture inhibits TXNIP-associated oxidative stress and inflammation to attenuate cognitive impairment in vascular dementia rats. CNS Neurosci Ther 24:39–46 29110407 10.1111/cns.12773 PMC6489958

[CR48] El-Ashmawy NE, Khedr EG, Alfeky NH, Ibrahim AO (2022) Upregulation of GLUT4 and PI3K, and downregulation of GSK3 mediate the anti-hyperglycemic effects of proanthocyanidins. Med Int 2:14 10.3892/mi.2022.39 PMC9829200 36698506

[CR31] El-Shaer NO, Hegazy AM, Muhammad MH (2023) Protective effect of Quercetin on pulmonary dysfunction in streptozotocin-induced diabetic rats via Inhibition of NLRP3 signaling pathway. Environ Sci Pollut Res 30:42390–42398 10.1007/s11356-023-25254-8 PMC10067641 36648717

[CR36] Fabre N, Rustan I, de Hoffmann E, Quetin-Leclercq J (2001) Determination of flavone, flavonol, and Flavanone aglycones by negative ion liquid chromatography electrospray ion trap mass spectrometry. J Am Soc Mass Spectrom 12:707–715 11401161 10.1016/S1044-0305(01)00226-4

[CR21] Flanagan E et al (2022) Chronic consumption of cranberries (Vaccinium macrocarpon) for 12 weeks improves episodic memory and regional brain perfusion in healthy older adults: A randomised, Placebo-Controlled, Parallel-Groups feasibility study. Front Nutr 9:849902 35662954 10.3389/fnut.2022.849902 PMC9160193

[CR39] Gudžinskaitė I et al (2020) Variability in the qualitative and quantitative composition and content of phenolic compounds in the fruit of introduced American cranberry (Vaccinium macrocarpon Aiton). Plants 9:1379 33081256 10.3390/plants9101379 PMC7602967

[CR47] Guo H, Dong Y-Q, Ye B-P (2016) Cranberry extract supplementation exerts preventive effects through alleviating Aβ toxicity in caenorhabditis elegans model of Alzheimer’s disease. Chin J Nat Med 14:427–433 27473960 10.1016/S1875-5364(16)30039-5

[CR43] H Z et al (2023) Protective Effect of Anthocyanins against Neurodegenerative Diseases through the Microbial-Intestinal-Brain Axis: A Critical Review. *Nutrients* 15 10.3390/nu15030496 PMC9922026 36771208

[CR33] Heid CA, Stevens J, Livak KJ, Williams PM (1996) Real time quantitative PCR. Genome Res 6:986–994 8908518 10.1101/gr.6.10.986

[CR15] Henriques JF, Serra D, Dinis TCP, Almeida LM (2020) The Anti-Neuroinflammatory role of anthocyanins and their metabolites for the prevention and treatment of brain disorders. Int J Mol Sci 21:8653 33212797 10.3390/ijms21228653 PMC7696928

[CR27] Hussain F et al (2017) Efficient hepatoprotective activity of cranberry extract against CCl4-induced hepatotoxicity in Wistar albino rat model: Down-regulation of liver enzymes and strong antioxidant activity. Asian Pac J Trop Med 10:1054–1058 29203101 10.1016/j.apjtm.2017.10.008

[CR44] Jakaria M et al (2019) Potential therapeutic targets of Quercetin and its derivatives: its role in the therapy of cognitive impairment. J Clin Med 8:1789 31717708 10.3390/jcm8111789 PMC6912580

[CR9] Khorkova O, Hsiao J, Wahlestedt C (2015) Basic biology and therapeutic implications of LncRNA. Adv Drug Deliv Rev 87:15–24 26024979 10.1016/j.addr.2015.05.012 PMC4544752

[CR17] Li X, Chen W, Xia J, Pan D, Sun G (2024) The effects of cranberry consumption on glycemic and lipid profiles in humans: A systematic review and Meta-Analysis of randomized controlled trials. Nutrients 16:782 38542695 10.3390/nu16060782 PMC10974925

[CR6] Liu L, Wang N, Kalionis B, Xia S, He Q (2022) HMGB1 plays an important role in pyroptosis induced blood brain barrier breakdown in diabetes-associated cognitive decline. J Neuroimmunol 362:577763 34844084 10.1016/j.jneuroim.2021.577763

[CR49] Luo L et al (2018) Protective effect of grape seed procyanidins against H2 O2 -Induced oxidative stress in PC-12 neuroblastoma cells: Structure-Activity relationships. J Food Sci 83:2622–2628 30221772 10.1111/1750-3841.14349

[CR37] McNab H, Ferreira ESB, Hulme AN, Quye A (2009) Negative ion ESI–MS analysis of natural yellow dye flavonoids—An isotopic labelling study. Int J Mass Spectrom 284:57–65

[CR62] Michailidis M et al (2022) Antidiabetic drugs in the treatment of Alzheimer’s disease. Int J Mol Sci 23:4641 35563031 10.3390/ijms23094641 PMC9102472

[CR18] Niesen S et al (2022) Fractionation of extracts from black chokeberry, cranberry, and pomegranate to identify compounds that influence lipid metabolism. Foods Basel Switz 11:570 10.3390/foods11040570 PMC8871205 35206045

[CR58] Park E, Choi H, Truong C-S, Jun H-S (2023) The Inhibition of autophagy and pyroptosis by an ethanol extract of Nelumbo nucifera leaf contributes to the amelioration of Dexamethasone-Induced muscle atrophy. Nutrients 15:804 36839161 10.3390/nu15040804 PMC9965294

[CR22] Pei H et al (2024) Preparation, characterization, bioactivity, and safety evaluation of PEI-modified PLGA nanoparticles loaded with polysaccharide from cordyceps militaris. Adv Compos Hybrid Mater 8:93

[CR19] Peixoto TC et al (2018) Cranberry (Vaccinium macrocarpon) extract treatment improves triglyceridemia, liver cholesterol, liver steatosis, oxidative damage and corticosteronemia in rats rendered obese by high fat diet. Eur J Nutr 57:1829–1844 28501921 10.1007/s00394-017-1467-2

[CR16] Pourshahidi S, Davari M, Anthocyanins (2020) Promising natural compounds for prevention and treatment of oral squamous cell carcinoma. Middle East J Rehabil Health Stud 7

[CR25] Ragab AE, Al-Madboly LA, Al-Ashmawy GM, Saber-Ayad M (2022) Abo-Saif, M. A. Unravelling the in vitro and in vivo Anti-Helicobacter pylori effect of Delphinidin-3-O-Glucoside rich extract from pomegranate exocarp: enhancing autophagy and downregulating TNF-α and COX2. Antioxid Basel Switz 11:1752 10.3390/antiox11091752 PMC9495706 36139826

[CR35] Rajbhandari R et al (2011) Determination of cranberry phenolic metabolites in rats by liquid Chromatography–Tandem mass spectrometry. J Agric Food Chem 59:6682–6688 21634376 10.1021/jf200673h PMC3165050

[CR2] Rom S et al (2019) Hyperglycemia-Driven neuroinflammation compromises BBB leading to memory loss in both diabetes mellitus (DM) type 1 and type 2 mouse models. Mol Neurobiol 56:1883–1896 29974394 10.1007/s12035-018-1195-5 PMC6320739

[CR59] Rousta A-M et al (2022) Therapeutic potential of Isorhamnetin following Acetaminophen-Induced hepatotoxicity through targeting NLRP3/NF-κB/Nrf2. Drug Res 72:245–254 10.1055/a-1792-2678 35359022

[CR5] Ruan Z, Li Y, Chen Y (2022) HECTD3 promotes NLRP3 inflammasome and pyroptosis to exacerbate diabetes-related cognitive impairment by stabilising MALT1 to regulate JNK pathway. Arch Physiol Biochem 1–12. 10.1080/13813455.2022.2093377 10.1080/13813455.2022.2093377 35913790

[CR8] Scassellati C et al (2021) Promising intervention approaches to potentially resolve neuroinflammation and steroid hormones alterations in Alzheimer’s disease and its neuropsychiatric symptoms. Aging Dis 12:1337 34341712 10.14336/AD.2021.0122 PMC8279527

[CR54] Shi C-S, Kehrl JH (2019) Bcl-2 regulates pyroptosis and necroptosis by targeting BH3-like domains in GSDMD and MLKL. Cell Death Discov 5:151 31839993 10.1038/s41420-019-0230-2 PMC6901440

[CR10] Shi Y et al (2019) Stabilization of LncRNA GAS5 by a small molecule and its implications in diabetic adipocytes. Cell Chem Biol 26:319–330e6 30661991 10.1016/j.chembiol.2018.11.012 PMC10498384

[CR14] Shimizu K et al (2019) Cranberry attenuates progression of Non-alcoholic fatty liver disease induced by High-Fat diet in mice. Biol Pharm Bull 42:1295–1302 31366865 10.1248/bpb.b18-00984

[CR13] Suriyaprom S et al (2022) Antioxidants of fruit extracts as antimicrobial agents against pathogenic bacteria. Antioxid Basel Switz 11:602 10.3390/antiox11030602 PMC8945554 35326252

[CR51] Tenenbaum L, Humbert-Claude M (2017) Glial cell Line-Derived neurotrophic factor gene delivery in Parkinson’s disease: A delicate balance between neuroprotection, trophic effects, and unwanted compensatory mechanisms. Front Neuroanat 11:29 28442998 10.3389/fnana.2017.00029 PMC5385337

[CR52] Torika N, Asraf K, Apte RN, Fleisher-Berkovich S (2018) Candesartan ameliorates brain inflammation associated with Alzheimer’s disease. CNS Neurosci Ther 24:231–242 29365370 10.1111/cns.12802 PMC6489976

[CR46] Vorhees CV, Williams MT (2006) Morris water maze: procedures for assessing Spatial and related forms of learning and memory. Nat Protoc 1:848 17406317 10.1038/nprot.2006.116 PMC2895266

[CR60] Wei Z, Jing Z, Pinfang K, Chao S, Shaohuan Q (2022) Quercetin Inhibits Pyroptosis in Diabetic Cardiomyopathy through the Nrf2 Pathway. *J. Diabetes Res.* 9723632 10.1155/2022/9723632 PMC9825227 36624860

[CR12] Xiao Y et al (2022) Urolithin A attenuates Diabetes-Associated cognitive impairment by ameliorating intestinal barrier dysfunction via N-glycan biosynthesis pathway. Mol Nutr Food Res 66:e2100863 35184377 10.1002/mnfr.202100863

[CR11] Xu W, Zhang L, Geng Y, Liu Y, Zhang N (2020) Long noncoding RNA GAS5 promotes microglial inflammatory response in Parkinson’s disease by regulating NLRP3 pathway through sponging miR-223-3p. Int Immunopharmacol 85:106614 32470877 10.1016/j.intimp.2020.106614

[CR3] Xue Y, Tuipulotu E, Tan D, Kay WH, C., Man SM (2019) Emerging activators and regulators of inflammasomes and pyroptosis. Trends Immunol 40:1035–1052 31662274 10.1016/j.it.2019.09.005

[CR28] Yadav SK, Pandey S, Singh B (2017) Role of Estrogen and Levodopa in 1-methyl-4-pheny-l-1, 2, 3, 6-tetrahydropyridine (mptp)-induced cognitive deficit in parkinsonian ovariectomized mice model: A comparative study. J Chem Neuroanat 85:50–59 28711564 10.1016/j.jchemneu.2017.07.002

[CR38] Yang L-L et al (2016) Pharmacokinetic comparison between Quercetin and Quercetin 3-O-β-glucuronide in rats by UHPLC-MS/MS. Sci Rep 6:35460 27775094 10.1038/srep35460 PMC5075792

[CR41] Yang Y, Zhao J-J, Yu X-F (2022) Expert consensus on cognitive dysfunction in diabetes. Curr Med Sci 42:286–303 35290601 10.1007/s11596-022-2549-9

[CR57] Ye T et al (2018) Gastrodin ameliorates cognitive dysfunction in diabetes rat model via the suppression of Endoplasmic reticulum stress and NLRP3 inflammasome activation. Front Pharmacol 9:1346 30524286 10.3389/fphar.2018.01346 PMC6262084

[CR56] Yu F et al (2022) Nitidine chloride induces caspase 3/GSDME-dependent pyroptosis by inhibting PI3K/Akt pathway in lung cancer. Chin Med 17:115 36175965 10.1186/s13020-022-00671-y PMC9524076

[CR20] Yung L-M et al (2013) Chronic cranberry juice consumption restores cholesterol profiles and improves endothelial function in ovariectomized rats. Eur J Nutr 52:1145–1155 22836513 10.1007/s00394-012-0425-2

[CR53] Zhang J, Tan H, Jiang W, Zuo Z (2014) Amantadine alleviates postoperative cognitive dysfunction possibly by increasing glial cell line-derived neurotrophic factor in rats. Anesthesiology 121:773–785 25251457 10.1097/ALN.0000000000000352 PMC4176814

[CR50] Zhang L et al (2017) Protective property of mulberry digest against oxidative stress - A potential approach to ameliorate dietary acrylamide-induced cytotoxicity. Food Chem 230:306–315 28407916 10.1016/j.foodchem.2017.03.045

[CR45] Zhao S, Zhang L, Yang C, Li Z, Rong S (2019) Procyanidins and Alzheimer’s disease. Mol Neurobiol 56:5556–5567 30649713 10.1007/s12035-019-1469-6

[CR4] Zheng Z, Li G (2020) Mechanisms and therapeutic regulation of pyroptosis in inflammatory diseases and cancer. Int J Mol Sci 21:1456 32093389 10.3390/ijms21041456 PMC7073143

